# Ethical Analysis of Egypt's Law Regulating Clinical Research

**DOI:** 10.1177/15562646221096188

**Published:** 2022-05-16

**Authors:** Amal Matar, Henry J. Silverman

**Affiliations:** 1 Centre for Research Ethics and Bioethics, Department of Public Health and Caring Sciences, 8097 Uppsala University; 2 12264 University of Maryland School of Medicine, Baltimore, Maryland, USA

**Keywords:** clinical trial law, informed consent, research ethics, ethical regulations, Egypt, low- and middle-income country

## Abstract

Lately, there has been increased research performed in Egypt. In response, the Egyptian Parliament published its first clinical research law in December 2020. The official version of the law was translated to English from Arabic and back by an accredited translation service. We performed an ethical analysis of the law based on the seven ethical requirements for clinical research proposed by Emanuel et al. and compared it with other regulations in the Arab region. The law contains provisions that fulfill all requirements for ethical research to varying degree. Provisions necessitating the sharing of participants’ data and biospecimens by the Central Intelligence Agency requires further specifications to ensure privacy protection. Also, the law poses problematic liabilities that could hamper medical research. Egypt's law compares favorably with other laws in the region. Potential items that require further specification can be addressed in the executive regulations currently being drafted for the law.

## Introduction

For almost two decades, multinational pharmaceutical companies have found Egypt to be an appealing place to outsource their clinical trials. Second to only South Africa, Egypt has the highest number of clinical trials being carried out by pharmaceutical companies, such as Roche and Novartis in Africa (Durisch, 2016; Zannad et al., 2019). Compared with other Arab countries in the Middle East, Egypt conducts the largest number of clinical trials and together with Saudi Arabia manages half of all clinical studies in the region (Silverman, 2017).

Several reasons explain Egypt's attraction as a clinical trial site. First, Egypt supports a suitable research infrastructure consisting of experienced researchers and other supporting personnel, e.g., clinical pharmacists. Second, Egypt has a population of almost 100 million inhabitants, most of whom are treatment naïve and hence, represent an ideal population to test out new drugs (Durisch, 2016; Silverman, 2017). Finally, Egypt lacks a strong regulatory body that is guided by national regulations, which might lead to inconsistent oversight. Accordingly, a few pharmaceutical companies might take advantage of this bureaucratic vacuum.

Before the issuance of the law, clinical research was regulated at the IRB and the Ministry of Health (MOH) levels. A study proposal is firstly submitted for approval by institution/department where the study will be conducted and its local IRB. After receiving these approvals, it is then reviewed by the Central Scientific and Research Ethics Committee at the MOH. If the study involves transferring biospecimens outside Egypt, authorization from the National Security Office at the MOH is also required (Saleh, 2017).

Egypt has established many Institutional Review Boards (IRBs) during the last ten years. There were no national ethical guidelines to guide IRBs, which resorted to international documents such as the Council for International Organizations for Medical Sciences (CIOMS), Declaration of Helsinki (DoH) and the Belmont report to guide their review process (Saleh, 2017). As of this writing there are more than fifty IRBs in Egypt. Furthermore, there is a network of IRBs managed by the Egyptian Network of Research Ethics Committees (ENREC). The network provides periodic trainings on GCP and Research Ethics to IRB members in Egypt and make available online resources such as Arabic templates for informed consent, a checklist for research review and training materials (Sleem, 2008). Together with Middle East Research Ethics Training Initiative, an NIH-sponsored program (Silverman et al., 2013), ENREC has promoted the enhanced ethical review of research in Egypt in the last decade. However, in contrast to other countries in the Arab Region, Egypt lacks formal regulations to ensure oversight of these IRBs as well as consistency in the review of research between the different IRBs (Silverman, 2017).

The first proposal for an Egyptian clinical trial law dates back to 2002 (Durisch, 2016), which was drafted by the Head of Education and Scientific Committee of the Egyptian Parliament. However, this draft law failed to gain approval of the Parliament. Another proposition surfaced in 2014 but was met with intense opposition from civil societies because it allowed clinical studies involving children, pregnant women and other vulnerable groups including prisoners and psychiatric patients without acceptable safeguards. It was rejected by the Pharmacists’ Syndicate and Members of Parliament who believed the law served primarily the interests of international pharmaceutical companies (Durisch, 2016).

On May 15, 2018, a new proposal was submitted and subsequently approved by the Parliament in August, 2020 (Harbi, 2020; Youssef, 2020). There were several driving forces that promulgated the passage of the law, several at the government level which included: The Ministry of Scientific Research, the Ministry of Health and the Ministry of Higher Education and Scientific Research, and several at the civil society level; the ENREC and other NGOs. With the input of the aforementioned organizations, three committees were entrusted to draft the law. These committees were comprised of individuals from various professions, researchers, legal experts, ethicists, and representatives from the State. In addition, the Head of the Parliament's Health Committee sought feedback from the Egyptian Coptic Church and Al-Azhar Institute (the Islamic authority in Egypt) (Abu Talib, 2018b). Despite cautious approval from civic organizations, criticisms against this proposed law came from medical faculty members and Muslim religious figures (Abu Talib, 2018a; Shabaan, 2018a, 2018b). The medical academics indicated several concerns with the proposed law. First, many embraced the view that the law should address only clinical trials and not all medical research. Second, the proposed penal penalties were severe and would discourage researchers from conducting medical research. In addition, the law mandated multiple regulatory entities (up to six) to review and approve clinical trials, a process that could prolong the onset of clinical trials for up to a year. Other concerns included: the absence of provisions regarding the protection of intellectual property; prohibiting the export of biological specimens to international collaborators; and lastly, lack of provisions for insuring and compensating research participants for research injury (Shabaan, 2018b). The religious figures also expressed strong views against the proposed law, calling it Islamically unlawful (haram) because a human body should not be exposed to “experiments” (Shabaan, 2018a, 2018b).

President Sisi embraced these concerns and also disapproved of several of the penal clauses; for example, the proposed articles prohibiting the exportation of tissue/genetic samples out of Egypt; the inclusion of Master and PhD students’ research under the scrutiny of the law; and exclusion of research institutes and other specialized research units under the Ministry of Education (Lasheen, 2018). As a result, the draft law was returned to Parliament for amendments and its revision required almost two years for the law to gain the approval of both the President and the Egyptian Parliament. Dar il-Iftaa (the highest Islamic ruling authority) in Egypt also endorsed the law to help combat Covid-19 pandemic (Badrawi, 2020) and some commentators claimed that the pandemic sped up the process of approval in order to allow vaccine trials in Egypt (AL-masry Al-youm, 2020).

The law was published in the official gazette on 23^rd^ December 2020 and was titled “Clinical Medical Research Regulation Law”. While several organizations and individuals from different professions provided important input to the development of this Law, we believe it would be useful to analyze how well the Law conforms to recognized ethical requirements for the review of research. The approach of using an ethical framework is reasonable insofar that other regulations originated from an ethical framework, for example, the U.S. regulations are based on the ethical framework of the Belmont Report. Our aim is to analyze the provisions of this law in relation to well-established ethical requirements for human subject research (Emanuel et al., 2000) and compare it with other regulations in the Arab region.

## Methods

The text of the law was translated into English from Arabic and then back translated by a certified translation service. The translation was later reviewed and adjusted by the first author.

We primarily used the guidance of Emanuel and colleagues regarding the ethical requirements of research (Emanuel et al., 2000) to direct our analysis of the Egyptian Law.

We also compared the Egyptian Law with other recent national regulations recently developed in the Arab Region of the Middle East.

## Results

**A. General Description of the law**

The law is composed of 12 chapters and 32 articles. Table 1 describes these sections.

**B. Analysis of the Law**

**Table 1.** Description of the Law and its Different Chapters.

Chapter	Title	Description
One	Definitions	Has one article that defines the terms used in the law, such as interventional medical research, preclinical research, research protocol etc.
Two	General Provisions	Has articles 2, 3, 4 & 5, which outline provisions on vulnerable populations, mandatory approvals for research and requirements of Egypt's GIA.
Three	The Supreme Council for Reviewing the Ethics of Clinical Research.	The two articles (6 & 7) detail the composition, role and responsibilities of the Supreme Council
Four	Institutional Review Boards and the Egyptian Drug Authority	Article 8 describes the role and responsibilities of IRBs and article 9 details Egyptian DA function in regards to clinical research.
Five	Phases of Clinical research and the Use of Placebos	Article 10 – describes the phases of clinical trials while article 11 details placebo use in clinical research.
Six	Research Participants Rights	Article 12 describes research participants' rights including provisions for obtaining an withdrawing informed consent, privacy protection. Articles 13 and 14 are provisions for enrolling and payment of research participants. Article 15 addresses handling of research participants' information and confidentiality of their data.
Seven	The Conditions, Procedures and Obligations Principal Investigator	This chapter has 4 articles dedicated to outlining the qualifications, roles & responsibilities of the PI and Co-PI
Eight	Obligations of the Sponsor of Research.	Articles 20 & 21 describe the roles, responsibilities as well as approvals required by sponsor to implement medical research.
Nine	Suspension and Early Termination of Medical Research	The previsions to suspend medical research is detailed in article 22 in this chapter.
Ten	Provisions for Using Human Samples for Medical Research	Provision for obtaining consent, storing and commercialization of human samples are outlined in article 23.
Eleven	Requirements of the Research Entity	It includes one article (24) which details the requirements for research entities to be able to conduct medical research. for example prohibition of conducting research in private clinics.
Twelve	Liabilities and Penalties	The chapter encompasses articles 25–32 that outlines liabilities in the event that those in charge of conducting medical research do not abide by the provisions of the law.

We present our results of the analysis of the law in the following paragraphs.

### Social Value and Scientific Validity

To protect against exploitation and waste of scarce resources, research must be evaluated for its social value to the community from where it is carried out. Scientific validity in terms of reliable and valid data generated from appropriate methods and statistical tools is required to ensure meaningful results (Emanuel et al., 2000). There are no definite clauses in the Egyptian Law that emphasize the requirement for considering the social value of research. However, the Supreme Council will be entrusted to setting ethical standards and regulations of medical research to protect humans, including the privacy of their samples and health data*,* The review by the Supreme Council would consider the national interest and international scientific developments (Chapter (ch) 3, article (art) 7 (2)).

In contrast, the provisions regarding scientific validity are more robust as it is emphasized in several sections of the law where compliance to Good Clinical Practice (GCP) is part of the roles and obligations of principal investigators (PIs) and research sponsors (ch 7 art 18 (2), ch 8 art 20 (3)). Also, the Supreme Council and Egyptian Drug Authority will inspect research facilities to ensure compliance to GCP (ch 3 art 7 (4) and 9 (4), respectively), while IRBs will monitor PIs and research sponsors (ch 4 art 8 (4)). Additionally, art 2 affirms that research should abide by the provisions of internationally recognized ethical standards and principles.

### Fair Selection of Study Population

To fulfill the ethical principle of justice, there should be the fair selection of research participants to guarantee that the disadvantaged and the vulnerable do not unfairly bear the burden of risky research, while the rich and socially advantaged reap the benefits of research (Emanuel et al., 2000).

Chapter 7 of the law delineates that it is the responsibility of the PIs to choose the research participants with complete impartiality. In addition, the law provides a clear definition of vulnerable populations deserving extra protections when they participate in research. Specifically, they are identified as research participants with restricted autonomy as a result of legal or cognitive or health constraints and thus are most susceptible to coercion or exploitation (Art 1–15).

The law details further safeguards that include restricting research involving vulnerable populations unless scientifically and ethically justified. For example, permitting studies that only address specific diseases within a vulnerable group and ensure obtaining informed consent for participation, either from the individual or his/her legal guardian. (Art 3).

### Favorable Benefits to Risks Ratio

Emanuel et al., give guidance regarding the identification and appraisal of potential risks in relation to potential research benefits. Benefits to research participants and the society should outweigh the potential harms expected from the study. Furthermore, researchers should undertake measures to minimize risks, provide safety monitoring and record adverse events when they occur. Such procedures will satisfy the ethical principles of beneficence and non-maleficence (Emanuel et al., 2000). Though the Egyptian law does not detail a procedure to evaluate the risks/benefits ratio, researchers are required to comply with international ethical requirements and good clinical practice.

In the Egyptian law, efforts to minimize risks include provisions requiring that both the PI and co-PI possess the necessary qualifications and expertise that enable them to carry out clinical trials. Moreover, they should be familiar with the ethical and scientific guidelines of medical research (Ch 7 art 16 (1)).

Furthermore, the law prohibits research participants from enrolling in more than one study simultaneously (ch 6, art 13). Finally, the Egyptian Drug Authority (EDA) is entrusted to evaluate all preclinical trials, scientific review of medicinal or biological products and revise documents related to test products in accordance with good manufacturing, storage, and distribution practices (ch 4 art 9). These provisions all contribute to minimizing risks.

### Independent Review and Institutional Review Boards

Accountability and managing conflict of interests are realized when research undergo independent review according to Emmanuel et al. This should be performed by an independent review board, whose members are not associated with the research being examined (Emanuel et al., 2000).

According to the law, clinical trials conducted in Egypt will have three levels of ethical and scientific oversight. The first is by local institutional review board (IRB) of research institutes or university hospitals (ch 4, art 8). The second level of oversight will be carried out by the Egyptian Drug Authority, which is entrusted with reviewing the scientific aspects of clinical trial protocols, the medical interventions whether biological, pharmaceutical or a medical device and relevant pre-clinical studies (ch 4, art 9). The final level of review is provided by the “Supreme Council for Reviewing the Ethics of Clinical Medical Research”.

The law stipulates the formation of the “Supreme Council” under the auspices of the Prime Minister and will constitute 15 members who will serve for four years. The council would have one or more representatives from the different ministries: Ministry of Health and Population, Ministry of Higher Education (where university hospitals and research institutes are affiliated), Ministry of Scientific Research, Ministry of Defense, Ministry of Interior, General Intelligence Agency, and the State Council. Representatives from the Interior and Defense are included because both ministries manage the Egyptian army and police hospitals, respectively, where clinical trials are conducted. In addition, three representatives of the public will be members of the supreme council (ch 3 art 6).

The Clinical trial law does not specify the composition of members for IRBs or of the Supreme Council. For example, there are no requirements for gender and ethnicity diversity and no requirements for continuing education in research ethics.

All clinical trials defined in the law are subject to review by the Supreme Council, which will also be responsible for creating a national database for medical research. Furthermore, all IRBs that review clinical research are obligated to register themselves at the Supreme Council.

There are no mandates regarding the independence of these three levels of oversight from their respective institutions. Furthermore, in the description of functions and operations of the IRB, EDA and the Supreme Council, there appears to be much overlap of responsibilities, such as ethical review and monitoring and inspection of clinical trials and research facilities. Moreover, the law does not specify accreditation procedures to any of the oversight bodies (Supreme Council or the EDA and IRBs).

Though chapter 3 addresses IRBs, there is little mention of its functions as well as lack of guidance regarding the criteria for the approval of research. The law also lacks mention of specific review pathways such as exempt, expedited, or full review research. Finally, the Law is silent regarding the disclosure and management of conflicts of interest.

### Informed Consent

The most crucial practice within research in order to satisfy a participant's autonomy is informed consent (Emanuel et al., 2000). The law requires informed consent in medical research (ch 1 art 1(21)) and it is defined as a written expression to which research participants voluntarily provide their consents via a signature or a fingerprint (in cases of participants who are analphabetic) after all aspects of research are explained including potential risks and benefits. If research participants cannot give consent, their legal authorized representative can provide it on their behalf.

Several aspects of informed consent are missing in the law. These include detailing necessary required elements of consent, such as purpose of the research, PI affiliations, source of funding, conflict of interests, right to withdraw from research, and return of research results. Furthermore, adapting consent procedures to accommodate the local culture (such as verbal consent or consulting family members) and adapting to individual information needs, are not elucidated within the law. Alternatives to written consent are not mentioned in the law, which are crucial in cases where a written consent can pose risks to research participants (for example domestic violence survivors). Moreover, the law does not pose requirements to ensure research participants’ comprehension of informed consent information, which is important in low-and-middle-income countries such as Egypt.

Research participants’ informed consent is required for using their bio-samples in medical research. For storage and future reuse of the biological samples, a prior informed consent is required from participants as well as the approval of the Supreme Council ch 10 art 23(2).

The law prohibits undue inducement of research participants, as form of cash, rewards and other benefits are prohibited except for reimbursement for transportation fees to and from study location and missed work time by participants in the study ch 6 art 14.

Research participants will be kept informed by the PI (and the research sponsor) of adjustments made to the research protocol, particularly if they pose safety risks ch 7 art 18(5). Finally, the law is vague about waiver of consent and lack provisions regarding children's enrollment in research or ways to obtain their assent.

### Respect for Persons

Respect for persons include protection of their privacy and assurances of the confidentiality of their data. Furthermore, participants should be allowed to withdraw from studies without punitive actions and be kept notified of evolving risks, benefits and results of their research (Emanuel et al., 2000).

Chapter 6 deals with privacy and confidentiality and stipulate protections of participants’ identity unless it is scientifically justified as appraised by IRBs and the Supreme Council. Also, approval is required from the participant or his/her legal guardian ch 6 art 12(2).

Finally, the law prohibits the publishing or marketing of research data or reports in different media, except after finalization of the study and obtaining approval from the relevant IRB, the Supreme Council and research participants ch 6 art 15(3).

However, research participant's data obtained during and after the completion of medical research will be made available to SC, EDA, local IRBs and General Intelligence Authority for auditing and review purposes as per ch 6, art 15(2). There are no clauses in the law that clearly states that research participants should be aware that their data will be shared with a law enforcement agent, such as General Intelligence Agency.

Chapter 6 deals with research related injuries, as the research sponsor is responsible to provide health insurance coverage for research participants to handle any injuries that may result from their participation in research. The insurance company must be an accredited company in the Arab Republic of Egypt and the contract should cover the duration of the study and up to one year after the conclusion of research. In case of research related injury, insurance provisions should cover the cost of treatment as well as the compensation for injury ch 8, art 20 (9, 10, 11).

Furthermore, under research participants’ rights, participants can withdraw from the research at any point in time without justification. However, the PI is required to inform them of potential harms ensuing from such withdrawal ch 6, art 12(1).

Moreover, there are adequate provisions that necessitate periodic monitoring of research and its supporting facilities (such as laboratories) to ensure compliance to research protocols as well as standards of good clinical practice (art 7 (4); art 8 (4); art 9 (4)). Furthermore, PIs are required to obtain approval from the local IRB, Supreme Council and the Egyptian Drug Authority to amendments made to the research protocol and communicate them to research participants, particularly if they expose them to higher risks art 18 (4,5).

With respect to monitoring the safety of research participants, the PI is entrusted to take actions to safeguard research participants’ dignity, physical and mental health and minimize side effects of the intervention or test drug. This include and is not limited to making necessary adjustments to the research protocol in event of severe side effects. In such situations, the PI is required to inform the research sponsor, the IRB, the SC and the EDA of the event and the measures taken to protect the study participants, within maximum 24 hours ch 7, art 18 (6).

The Law, however, fails to elucidate transparency measures such as declaring conflicts of interests by members of the Supreme Council, Egyptian Drug Authority, and IRBs in the ICF.

## Discussion

The new Egyptian clinical trial law fulfills most of the ethical requirements for medical research as prescribed by Emanuel and colleagues (Emanuel et al., 2000, 2004). Egypt is the fourth country in the Arab region to issue a law regulating clinical research. It is preceded by Jordan, which was the first country to publish a clinical research law in 2001 (Ramahi & Silverman, 2009) (an update was issued in 2011 by Jordan's Food and Drug Administration, 2011). This was followed by the Kingdom of Saudi Arabia in 2010 issuing “*the Law of Ethics of Research on Living Creatures*” (Alahmad, 2017). Lastly, Morocco passed its law “*Protection of Persons Participating in Biomedical Research*” (Law Number 28–13), in 2015 (Adarmouch, 2017). Upon analysis, all these laws succeed in satisfying many of the ethical requirements of research with varying degrees.

The Egyptian law has explicit stipulations to protect vulnerable populations deserving extra protections. Characteristics attached to being vulnerable include limited cognitive ability, marginal health status, and the lack of decision-making capacity to give a valid, informed consent. The Law, however, does not include other characteristics usually attached to the vulnerable state. These include economic status, level of education, and dependent relationships, e.g., prisoners and children (Emanuel et al., 2004).

Justifications for involving individual with vulnerabilities includes a “necessity” requirement insofar that the research investigates diseases that are unique to the vulnerable group. Special protections include the requirement of consent of a legal representative for adults who lack capacity and the consent of both parents when enrolling children in research. Adequate provisions to define and protect vulnerable groups are present in all other laws in the Arab countries except for the Jordanian law (Ramahi & Silverman, 2009).

Another significant aspect of the Law includes several layers for monitoring of clinical research and inspection of facilities, where they are being conducted. This would be carried out by local IRBs, the Supreme Council, and the Egyptian Drug Authority and by research sponsors. Furthermore, there are adequate provisions to require research sponsors to have insurance for study participants in case of research injury and the provision of medical care during the trial and after its completion, if necessary. Furthermore, the sponsor is obligated to compensate and treat participants who experience research injury. These requirements are also echoed in both the Moroccan (Adarmouch, 2017) and the Jordanian laws (Ramahi & Silverman, 2009). The Saudi law stipulates the formation of a Monitoring Office to oversee local IRBs work and address grievances posed by researchers and research participants (Alahmad, 2017).

Other provisions that support respect of persons include prevention of undue inducement and prohibition of a subject's multiple concurrent enrollments in clinical trials. These measures guard against creating a category of patients that would make enrollment in clinical trials as a form of livelihood. The Moroccan law has a similar stipulation that forbids a research participant engaging in more than one study. Indeed, it necessitates that a study protocol specifies a “grace period” before one can enroll in a new trial. The Moroccan also limits payments to reimbursement of costs endured by research participants (Adarmouch, 2017).

The Law also addresses the utilization of human samples in medical research. This includes a comprehensible definition of the term and followed by clauses that permit future research after acquiring Supreme Council's authorization and subjects’ consent. However, further clarification is needed regarding a definition of research and human subject research especially for biospecimens that are de-identified. Such clarification would be helpful in regard to the requirement of informed consent in use of biospecimens in secondary research.

The law provides an explicit prohibition of commercialization of human samples obtained for medical research. This can prove challenging in cases of developing stem cells and gene therapies, where human samples are the raw material. In addition, a security clearance from GIA is required to transport samples abroad. The Moroccan Law explains measures of consent for donating human samples to biobanks and requires that foreign collaborators have “local representatives” (Adarmouch, 2017). Similarly, the Saudi law has clear provisions on definition of human samples and how to handle them as well as stipulated conditions for transport outside KSA (Alahmad, 2017). The Jordanian law lacks such definitions and requisites (Ramahi & Silverman, 2009).

The Law provides for the establishment of the “Supreme Council” that will be entrusted with setting standards and policies to review all clinical trials and multi-national studies. It is unclear if the SC will function as an IRB at a national level or has a distinctive function of its own. There are no requirements that its members should have knowledge and expertise in bioethics or clinical research. The membership of the committee ensures representation from all entities that perform clinical trials in Egypt including representatives from Ministry of Defense and Interior. A centralized committee model also exists in Jordan as the Clinical Studies Committee within Jordan FDA (Ramahi & Silverman, 2009), as a Central Research Ethics Committee for each of Morocco's regions (Adarmouch, 2017) and lastly as the National Committee of Bioethics in Saudi Arabia (Alahmad, 2017).

It appears that many members of the Supreme Council will comprise of individuals representing law enforcement and the military. For example, almost 16 of Egypt's 27 governorates have at least one army hospital. In Cairo and Alexandria, two of the most populated cities in Egypt, there are 27 army hospitals and clinics, which function under the auspices of Ministry of Defense. They provide services to army officials, their families and soldiers doing their compulsory military service (Egypt’s Ministry of Defense, 2018). Likewise, there are several police hospitals that serve police officers, their families, and members of Ministry of Interior. One can easily foresee that medical research in these entities pose their own set of ethical challenges, such as the hierarchy and dependent relationships of different army officials. The positive aspect within this provision is medical research in those hospitals will be under SC scrutiny and must comply to ethical and scientific standards as its civilian counterparts.

We have flagged a few concerns within the law. One includes the obligation to share research participants’ data with the Egyptian General Intelligence Authority after completion of clinical trials (art 15(2)). Such sharing represents a concern with data confidentiality as it allows law enforcement agencies to access highly sensitive information. Moreover, there is no mention that the access of such data will be disclosed to potential research participants. Mention of such data access might discourage participation in clinical trials and hence, there should be recommendations of when these ministries can access private information to allay such concerns. The published ethical analyses of the other three laws do not have corresponding requirements.

The provisions for liabilities and penalties appear to be overly severe and could discourage researchers, research sponsors and research institutes from partaking in medical research (Saleh et al., 2021). For example, there are clauses of hard labor-imprisonment (up to ten years) and large fines (up to half a million Egyptian pounds) in cases of breaching the terms of the law if these resulted in severe sides effect, disability or death. The fines and imprisonment are to be multiplied according to number of research participants affected (ch 12 art 25–32). But the Egyptian law is not unique in this respect, as there are comparable articles describing prison sentences and/or large monetary fines in all other three laws (Adarmouch, 2017), (Ramahi & Silverman, 2009) with varying degrees of severity, KSA being the least stringent (Alahmad, 2017). One rationale is to ensure enforceability and compliance to the laws by stakeholders who function unrestricted in a relatively deregulated field.

The Egyptian law is selective regarding the phases of clinical trials that can be performed within the country. All phases of clinical trials (1–4) are allowed if the drug to be tested is made in Egypt. If drugs are manufactured outside Egypt, only phase 3 and 4 trials are permitted. The exception being if drugs target only locally endemic and rare diseases then phase 2 clinical can be granted approval (ch 5 art 10). These provisions may enhance building local capacities and competencies but may exclude therapies that involve advanced technology, which are too expensive, require time and special expertise to develop and therefore not readily available in a LMIC such as Egypt. However, this may persuade research sponsors and international collaborators to spend on local infrastructure and build local competencies as has been reiterated in some of the recent literature (Serwadda et al., 2018).

Though the general ethical requirements are broadly fulfilled by the law, there are several deficiencies. For example, a waiver of consent is not mentioned for research in the emergency setting. This is particularly relevant during the covid-19 pandemic, where extensive research is being conducted in critical care units. Waiver of consent is also not mentioned for retrospective record review. Moreover, the law is silent on transparency measures such as disclosing and managing conflict of interests for members of SC or IRB or stating their institutional affiliations, or ensuring independence of IRBs and SC.

Relating to IRBs, more details are needed regarding its operations, member composition and pathways of reviews (exempt, expedited and full board). Likewise, no provisions are provided regarding community engagement in research or culture appropriation of some of the ethical requirements, for example informed consent procedures and accepting verbal consent in the presence of a witness. This is particularly important for research addressing “taboo” concerns such as HIV or domestic violence. Moreover, Egypt has a wide difference in rural versus urban cultural norms. Minority groups such as Nubians and Bedouins would benefit with guidance regarding community engagement measures, which would reduce misunderstandings as well as ensure they are not left behind in the research endeavor. Lastly, there is little mention regarding return of results especially in context of international research.

The Declaration of Helsinki (DoH) provides requirements for complying to ethical dissemination and publication of results, which are overlooked in all Arab laws, particularly aspects of authorship, plagiarism, and reporting of results. These issues are of particular concerns due to the exponential growth of predatory journals and lack of awareness among researchers of the LMICs (Xia et al., 2015). Furthermore, the DoH addresses measures for animal welfare and environment (article 11: consideration for the environment and article 21: animal welfare) (World Medical Association, 2013), which have not been referred to in the Egyptian law. In contrast, there are stipulation regarding animal research in the Saudi law (Alahmad, 2017). Another potential advantage is that the Saudi law complies to Islamic Sharia prerequisites (Alahmad, 2017), which makes it relevant and a good reference to address some of the objections posted by Egyptian Muslim scholars, who called for prohibiting the Egyptian law (Shabaan, 2018a). Having said that, precautions should be taken to respect other religious minorities' views in the country.

The law lacks a definition of human subject research, which is important for rapidly growing biospecimen research and biobanking endeavors in Egypt (Abd El-Aal et al., 2016). The concern is that samples when de-identified would not meet the definition of a human subject, and as such would not fall under the category of clinical research per the provisions of the law and would not require ongoing ethics review. Alternatively, as per the definition in the law (ch 1 art 17), all types of samples regardless of their degree of deidentification and sensitivity would be required to be reviewed by SC according to the law. Thus, the lack of definition can lead to under scrutiny or over scrutiny by SC. Lastly, including exempt and expedited categories in the law would greatly benefit the research review process of social sciences and humanities studies such as surveys, interview, and educational research. The review process for these types of studies, according to the law, would be completed by the full board of the IRB at the research institution and need not be referred to the Supreme Council.

The new law can be viewed as a shift from self-regulation to a more centralized national directive (Strode, 2015). Previously, IRBs operating within the local research and research institutes and the MOH were entrusted to review research and ensure compliance to international standard documents such as DoH or CIOMS (Matar & Silverman, 2013; Saleh, 2017; Silverman et al., 2013; Sleem, 2008). The law, it can be argued, would harmonize the fragmented regulatory scene to a more centralized and coordinated approach. This, however, does not dispel the fears of increasing the complexity and bureaucratic process of clinical research review. Particularly, since there seems to be three levels of scrutiny within the law: the SC, the EDA, and the IRB, with overlap of responsibilities and authority and consequently the addition of unnecessary toll of red tape. Furthermore, lack of guidance on matters of conflict of interest and disclosure can jeopardize the ethical review of research.

## Conclusions

The Egyptian Law succeeds well regarding conforming to the ethical requirements for the review and oversight of research, as developed in the framework of Emanuel and colleagues. The Law is also similar in this regard to other laws in the Arab region. Furthermore, it considers unique local settings such as the presence of potentially research-active Army and Police Hospitals, presence of private medical clinics and over representation of vulnerable population. Likewise, the composition of the Supreme Council represents the different actors actively contributing to medical research in Egypt. We recommend that representatives from these agencies be selected based on their medical and research expertise rather than their law enforcement or military competency. As the implementation of the Law can be open to various interpretations, we recommend the establishment of a National Research Committee to provide ethical guidance on its application.

### Best Practices

In Egypt, our findings can be beneficial to writing the executive regulations of the law, which are currently being drafted. Furthermore, our analysis of the results might be helpful for other LMICs undertaking similar endeavors. Specifically, the drafters of the regulations can clarify vague or missing dimensions, such as regulations involving children and prisoners in research. We recommend that each country consider the local setting and ensure that their regulations are relevant to the national research context and needs while respecting the requirements for ethical research.

### Research Agenda

Further research is warranted to compare this law to regulations in other LMICs such as India, South Africa, and High-Income Countries in Europe and the US. In addition, it would be of value to assess the impact of the law on the ability of researchers to implement their research, enhancement of the review process by Egyptian IRBs, and the effect on attracting international clinical research in Egypt. Likewise, research on the activities and challenges faced by the Supreme Council can be another way to evaluate the effect of the law on Egypt's medical research. However, both types of research would need a few years of implementation before effects are discernable.

### Educational Implications

Using Emanuel et al., criteria for ethical research as the framework against which laws and regulations are being assessed can be a valuable tool for researchers, lawyers, and research ethicists. It has already been used in evaluating other laws and regulations in the Arab region (Adarmouch, 2017; Alahmad, 2017; Ramahi & Silverman, 2009). Moreover, the method can be taught to policymakers, lawyers, and researchers collaborating to draft laws and regulations on research in other LMICs.

In the Egyptian context, the tool can train members of the Supreme Council on research ethics. It can be further customized as a framework to evaluate research submitted to the Supreme Council.

## List of abbreviations

art: article
ch: chapter
EDA: Egyptian Drug Authority
ENREC: Egyptian Network for Research Ethics Committees
GIA: General Intelligence Agency
GCP: Good Clinical Practice
HIV: Human immunodeficiency virus
IRB: Institutional Review Board
KSA: Kingdom of Saudi Arabia
LMICs: Low-middle-income countries
MERETI: Middle East Research Ethics Training Initiative
PI: Principal Investigator
REC: Research Ethics Committee
SC: Supreme Council
